# Utilisation of primary healthcare services by patients with hypertension before, during and after the COVID-19 pandemic in Turku, Finland—are digital services creating disparity?

**DOI:** 10.1177/14034948251392076

**Published:** 2025-11-12

**Authors:** Jouni K. Johansson, Päivi Korhonen

**Affiliations:** University of Turku, Department of General Practice and The Wellbeing Services County of Southwest Finland, Turku, Finland

**Keywords:** Epidemiology, hypertension, primary healthcare, healthcare services, cardiovascular diseases, quality in healthcare, utilisation of healthcare service, follow-up, pandemic

## Abstract

**Aims:**

The objective of the study was to assess differences in the accessibility of general practitioner (GP office visits, telephone contacts and electronic communication for patients with hypertension before, during and after the COVID-19 pandemic in primary healthcare.

**Methods:**

From the primary healthcare register of the city of Turku in Southwestern Finland, subjects with the diagnosis of hypertension were identified. Data of GP office visits, telephone contacts, electronic communication, concomitant cardiometabolic diseases, low density lipoprotein and glomerular filtration rate were gathered from the hypertensive patients. The follow-up period was from 2019 to 2022.

**Results:**

The number of patients with hypertension decreased between the years 2019 and 2020 and again increased in 2021 to the 2019 level. Electronic communication increased steadily from 2019 to 2022, whereas telephone contacts decreased. A higher number of telephone contacts and higher number of electronic communication were associated with higher number of office visits. Elderly subjects used less electronic communication than younger subjects and the difference grew larger during and after the COVID-19 pandemic.

**Conclusions:**

**Elderly subjects with hypertension use digital services in primary healthcare less than their younger counterparts. Digital technologies may create structural inequality in access to, and availability of, primary healthcare services.**

## Background

Hypertension is potentially hazardous and highly prevalent risk factor in the general population. High systolic blood pressure (BP) is the leading risk factor for mortality, accounting for 10.8 million deaths globally in 2019 [1]. According to the Finnish national health examination survey in 2017, the prevalence of hypertension is 43% among the adult population [2]. Primary healthcare is responsible for the treatment of most patients with hypertension and, thus, access to healthcare services is crucial to control this major public health problem.

The COVID-19 pandemic challenged the capacity of healthcare systems to provide adequate care for patients with long-term diseases and conditions. A review of 81 studies across 20 countries reported that healthcare utilisation decreased by about one-third during the pandemic, with the highest reductions of 42% for office visits [3]. However, digital tools were increasingly utilised instead of office visits to help patients to connect with their healthcare providers. At the same time, it became apparent that utilisation of digital healthcare was difficult, especially among the elderly and ethnic minorities [4].

The International Society of Hypertension position paper in 2022 summarized the lessons from the COVID-19 pandemic and concluded that virtual care of chronic diseases including hypertension will continue in some form into the future. Moreover, further investigation is needed in delivering telehealthcare in specific populations such as the elderly or people with poor health [5].

## Aims

Currently, it is unknown how the COVID-19 pandemic has affected the utilisation of primary healthcare services among patients with hypertension. The objective of the present study was to assess the differences in accessibility of general practitioner (GP) office visits, telephone contacts and electronic communication for patients with hypertension in the primary healthcare centres of the city of Turku, Finland. An estimation of the backlog of hypertension treatment caused by the pandemic was also performed by comparing the amount of healthcare services utilised before, during and after the COVID-19 pandemic.

## Methods

### Study population

Subjects diagnosed with hypertension and at least one prescription renewed during the follow-up period of 1 January 2019–31 December 2022 were identified from the primary healthcare register database of the city of Turku (192,962 inhabitants in 2019), Finland.

In Finland, prescriptions can be made for the maximum of 2 years. This practice has been in use since 1 January 2017; previously, prescriptions could be made for a maximum of 1 year.

The number of face-to-face office visits, telephone consultations and electronic communication (text and web-based messaging) to GPs were collected from the database. Data, including the patients’ age, sex, antihypertensive medication usage, comorbidities (diabetes, coronary artery disease and atrial fibrillation) using the tenth version of the International Statistical Classification of Disease (ICD-10) diagnostic codes, and levels of low-density lipoprotein cholesterol (LDL-C) and estimated glomerular filtration rate (eGFR) were gathered. In case of multiple measurements of LDL-C and eGFR during the follow-up period, the mean of all values available was used. In primary care units, the diagnosis of hypertension (ICD-10 code I10) was made by the physician based on the current hypertension diagnostic guidelines.

In Finland, most patients with hypertension are diagnosed and treated in primary care. Only patients with diagnostic uncertainty or resistant hypertension are referred to secondary care internal medicine clinics. Hypertension treatment in accordance with the treatment recommendations entitles a patient to the basic reimbursement for BP medication (40%).

### Definitions

Registered telephone consultations were calls from a GP to a patient. Electronic communication included one-way text messages from a GP to a patient (a patient could not answer the text message) and web-based messaging (secured two-way text-based messaging integrated to an electronic health record (EHR), attaching files is not currently possible). Using web-based services/messaging requires a strong authentication from the patients’ side.

The total number of office visits, telephone consultations and electronic communication contacts were summarised per each year. Statistical data about electronic communication was available reliably from 2019 onwards.

### Study period

For data collection, the cut-off dates defining a year were from 1 January to 31 December.

From the year 2018, electronic communication methods have been recorded systematically into EHRs in Turku.

On 16 March 2020, the Finnish government declared a state of emergency in the country to slow down the spreading of COVID-19 [6]. In the healthcare centres of Turku, a few GPs were reassigned to COVID-19-related tasks.

Since mid-May 2020, extensive restrictive measures were eased gradually, but social distancing and lack of healthcare personnel still hindered follow-up visits of patients with chronic conditions such as hypertension-related diseases.

In April 2021, COVID-19 mass vaccinations for all citizens aged 18 years or more began and, by the end of March 2022, 65% of people aged 70 years and over were triple-vaccinated [7]. The year 2022 can be regarded as the year when primary healthcare of the city of Turku returned to prepandemic levels.

### Statistical analysis

Database management and statistical analysis were performed with SAS 9.4 (SAS Institute, Cary, NC). *P* values less than 0.05 were considered statistically significant.

Chi-squared test was used to compare differences across multiple categorical variables during follow-up years. Linear regression models were used to compare the differences between continuous variables during follow-up years. Multivariate linear regression model analyses (using proc GLM statement) were performed (see Table III) to identify the factors associated with the healthcare service use. The covariants included were sex, age, number of antihypertensive medication, number of telephone contacts, number of electronic communication, presence of diabetes (yes/no), presence of coronary artery disease (yes/no) and presence of atrial fibrillation (yes/no). The covariants were selected according to clinical significance. The correlations between office visits (2019, 2020, 2021 and 2022) and the covariant were statistically significant (*P* < 0.05), with the exception of age and sex, which were not statistically significant.

Age was divided into tertiles to show the age-related differences in the amount of office visits to GP, telephone contacts and usage of electronic communication (Figure 2).

β values for the model parameter estimates were calculated to quantify the effect of each independent variable on the dependent variable (office visits) (see Table III).

## Results

In 2019, the year before the COVID-19 pandemic, there were altogether 22,260 patients with a diagnosis of hypertension and at least one drug prescribed in the register database of primary healthcare centres of the city of Turku. In 2020, when social distancing was at its height, there were fewer patients with hypertension (1300 (6%)) who contacted healthcare centres. Thereafter, the total amount of subjects with hypertension increased to the 2019 level. The mean age of patients and their mean LDL-cholesterol level decreased slightly, but their mean eGFR remained fairly constant. The ratio between men and women remained constant. The proportion of hypertensive subjects with diabetes, coronary artery disease or atrial fibrillation contacting healthcare units decreased slightly during follow-up, from 36% to 35%, from 17% to 15% and from 19% to 17%, respectively (Table I).

**Table I.** Population characteristics.

Variable	2019	2020	2021	2022
Hypertensive patients, n^a^	22,260	20,960	22,992	22,427
Men	42%	42%	43%	42%
Mean age, years (SD)	74 (11)	73 (11)	73 (12)	73 (12)
Glomerular filtration rate, ml/min/1.73m^2^ (SD)^b^	71 (19)	72 (19)	73 (19)	72 (19)
LDL cholesterol, mmol/l (SD)^c^	2.7 (0.9)	2.5 (0.9)	2.5 (0.9)	2.4 (0.9)
Diabetes	36%	36%	35%	35%
Coronary artery disease	17%	16%	15%	15%
Atrial fibrillation	19%	18%	17%	17%

eGFR, estimated glomerular filtration rate; ICD-10, tenth version of the International Statistical Classification of Disease; LDL-C, low-density lipoprotein cholesterol.

a Patients with hypertension diagnostic code (ICD-10: I10) and at least one prescription of any drug during the calendar year.

b GFR was measured in 72–80% of the subjects in 2019–2022.

c LDL was measured in 58–63% of the subjects in 2019–2022.

In Turku city there were 1.2 (year 2019), 1.1 (year 2020), 1.1 (year 2021) and 1.0 (year 2022) outpatient medical visits (physicians) in primary healthcare per inhabitant. In inhabitants aged 65–74 years there were 2.6 (2019), 2.5 (2020), 2.3 (2021) and 2.3 (2022) outpatient medical visits.

In inhabitants aged 75–84 years, there were 3.3 (2019), 2.9 (2020), 2.7 (2021) and 2.7 (2022) outpatient medical visits. The corresponding outpatient medical visits in inhabitants aged 85 years and over were 2.9 (2019), 2.7 (2020), 2.7 (2021) and 3.0 (2022) per inhabitant.

The corresponding outpatient medical visits of hypertensive subjects are presented in Table II.

**Table II.** Office visits of subjects with hypertension on average per study year.

Study year	2019	2020	2021	2022
Age group, years	Office visits			
65–74	4	3.4	5.2	4.1
75–84	4.3	3.7	5.4	4.2
>85	4	3.9	5.2	3.5

The GP visits of subjects with hypertension were fourfold higher compared with outpatient medical visits of the Turku inhabitants in general.

### Methods to contact GP

**Figure 1.**
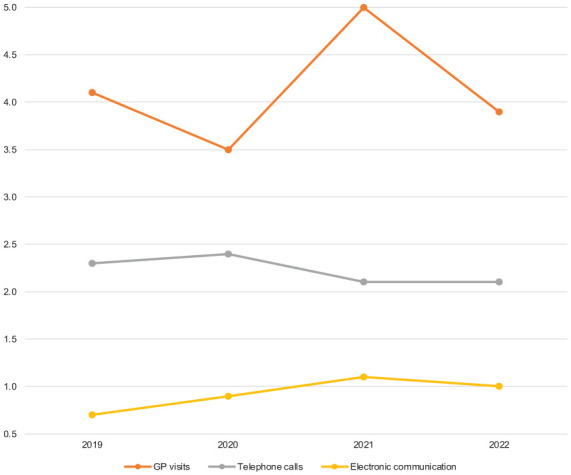
Use of healthcare service by communication method. Points indicate mean number of GP visits, telephone contacts and electronic communication of all hypertensive subjects in 2019, 2020, 2021 and 2022.

Office visits to GP by subjects with hypertension decreased slightly between 2019 and 2020, but increased thereafter in 2021, *P* < 0.01 for all (Figure 1).

Office visits of multimorbid hypertensive subjects with or without diabetes decreased by 11% between 2019 and 2020, and those of multimorbid hypertensive subjects with coronary artery disease or atrial fibrillation decreased by 15% between 2019 and 2020 (*P* < 0.0001 for all).

Electronic communication increased steadily from 2019 to 2022. There was a statistically significant difference between 2019 and 2021 (*P* < 0.0001), whereas no significant difference was detected between 2021 and 2022 (*P* = 0.09).

**Figure 2.**
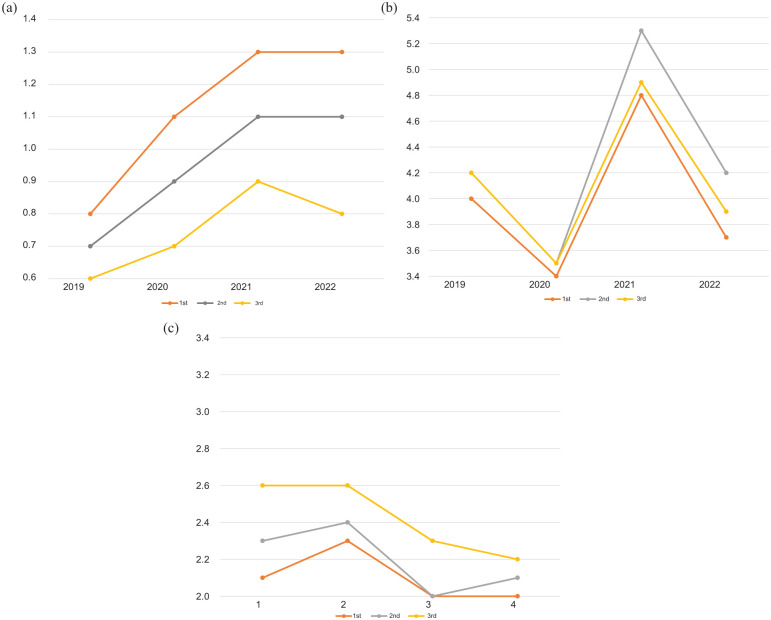
Use of electronic communication by patients with hypertension according to tertiles of age. Points indicate mean numbers of electronic communication, GP visits and telephone contacts per subject. Mean age was 60–61 years in the first tertile, 74–75 years in the second tertile and 84–86 years in the third tertile.

Telephone contacts decreased during the years 2019–2021 (*P* < 0.0001), but not between 2021 and 2022 (*P* = 0.95) (Figure 2).

### Ageing and utilisation of healthcare services

Elderly subjects (mean age 84–86 years) used electronic communication less often than younger subjects (mean age 60–61 years) and the difference grew larger during and after the COVID-19 pandemic (Figure 2) (*P* < 0.0001 between the first and third tertile in all follow-up years).

In addition, elderly subjects (mean age 84–86 years) had fewer office visits between 2021 and 2022 than their younger counterparts (mean age 74–75 years) but more than the subjects in the lowest tertile of age (mean age 60–61 years) (*P* < 0.0001 between the second and third tertile in all follow-up years) (Figure 2).

Elderly subjects (mean age 84–86 years) had more telephone contacts than their younger counterparts (mean age 74–75 years) or younger subjects (mean age 60–61 years) (*P* value between <0.0001 and 0.006 between the first and third tertile, and *P* < 0.0001 between the third and second tertile), except in the year 2022, where no difference was detected between older subjects and their younger counterparts (*P* = 0.70; Figure 2).

### Factors associated with higher number of GP office visits

Higher number of telephone contacts and higher number of electronic communications were associated with higher number of office visits in the general linear models during all follow-up years (Table III). Lower age and coronary artery disease were associated with higher number of office visits in 2019. Factors included in the model explained between 11% and 14% of the number of office visits.

**Table III.** Factors associated with healthcare service use.

	β	SE	*P*
Office visits in 2019			
Male sex	0.04	0.07	0.6
Age	–0.01	0.003	0.0001
Number of antihypertensive medications	–0.007	0.03	0.8
Telephone contacts	0.6	0.01	<0.0001
Electronic communication	0.2	0.02	<0.0001
Diabetes	–0.05	0.07	0.5
Coronary artery disease	0.2	0.09	0.01
Atrial fibrillation	–0.09	0.09	0.3
*R*^2^ (%)	12		
	β	SE	*P*
Office visits in 2020			
Male sex	0.1	0.07	0.05
Age	–0.005	0.003	0.1
Number of antihypertensive medications	–0.04	0.03	0.1
Telephone contacts	0.5	0.01	<0.0001
Electronic communication	0.2	0.02	<0.0001
Diabetes	–0.1	0.07	0.05
Coronary artery disease	0.2	0.09	0.06
Atrial fibrillation	0.0006	0.09	1
*R*^2^ (%)	11		
	β	SE	*P*
Office visits in 2021			
Male sex	0.2	0.06	<0.0001
Age	–0.002	0.003	0.4
Number of antihypertensive medications	0.04	0.03	0.1
Telephone contacts	0.6	0.01	<0.0001
Electronic communication	0.2	0.01	<0.0001
Diabetes	0.02	0.06	0.7
Coronary artery disease	0.01	0.08	0.9
Atrial fibrillation	–0.1	0.08	0.2
*R*^2^ (%)	14		
	β	SE	*P*
Office visits in 2022			
Male sex	0.23	0.06	0.0002
Age	0.004	0.003	0.16
Number of antihypertensive medications	–0.04	0.03	0.18
Telephone contacts	0.5	0.01	<0.0001
Electronic communication	0.3	0.01	<0.0001
Diabetes	–0.03	0.06	0.6
Coronary artery disease	0.2	0.08	0.05
Atrial fibrillation	0.04	0.08	0.6
*R*^2^ (%)	14		

Multivariate regression models were performed using general linear regression models, using SAS 9.4, proc GLM statement. The covariants included were sex, age, number of antihypertensive medications, number of telephone contacts, number of electronic communications, presence of diabetes (yes/no), presence of coronary artery disease (yes/no) and presence of atrial fibrillation (yes/no). β values for the model parameter estimates were calculated to quantify the effect of each independent variable on the dependent variable (office visits).

## Discussion

Overall GP visits of subjects with hypertension were fourfold higher compared with GP visits of Turku inhabitants in general.

This study showed that GP office visits by subjects with hypertension contacting primary healthcare unit decreased slightly between the years 2019 and 2020, but increased thereafter in 2021. Electronic communication increased steadily from 2019 to 2021, reaching a plateau in 2022, whereas telephone contacts decreased. Elderly subjects (mean age 84–86 years) used less electronic communication but more telephone contacts than younger subjects (mean age 60–61 years) and the difference grew even larger during and after the COVID-19 pandemic (Figure 2). The more patients used electronic or telephone contacts, the more they also had office visits with GPs. This raises the question of whether patients familiar with digital health services gain easier access to GP visits as well.

The GP visits of subjects with hypertension were about 1.2- to 2.2-fold higher compared with subjects in the same age group, meaning that subjects with hypertension had a more intense contact to primary healthcare than inhabitants of Turku city in general who used primary healthcare services. In addition, older subjects with hypertension (>85 years) had fewer GP visits than their younger counterparts (subjects with hypertension aged 65–74 years). This may indicate that aged people are in a vulnerable position in relation to accessing primary healthcare services.

The present study indicates that healthcare services during the COVID-19 pandemic increased after a small initial decline among patients with hypertension in primary care. In reality, offerings of healthcare services transformed rapidly towards digital services, which probably explains the trend.

The number of email contacts between GPs and enlisted patients more than doubled during the pandemic also in Denmark, and there was no reduction in contacts among patients with cardiovascular disease or type 2 diabetes [8]. Another Danish study reported that GPs welcomed telehealth solutions at the beginning of the COVID-19 pandemic, but their willingness to use these options generally diminished when face-to-face consultations were considered possible again [9].

The COVID-19 epidemic landed in Finland late compared with many other countries, and thus the authorities had time to learn from the experiences from other countries. The numbers of infected and dead were quite low in Finland compared with many other countries. The instructions on physical distancing were well obeyed, especially by elderly citizens who were not used to digital services.

In Turku, the population with hypertension decreased in 2020. This is probably due to physical distancing measures aimed at slowing the spread of the virus. Accordingly, Finland experienced no excess mortality in 2020 but exhibited highest excess mortality in 2022 compared with the prepandemic period [10].

During the 4-year follow-up in our study, the difference in use of electronic communication broadened between elderly subjects with hypertension and their younger counterparts. This may lead to increased structural inequality in the accessibility of primary healthcare services (Figure 2). In Finland, the prevalence of hypertension is more than 80% among people aged over 70 years [2]. Using web-based communication requires strong authentication by the patients, and therefore requires also suitable digital equipment, internet access and some knowledge of digital health services from the patient. This requirement might cause some, especially elderly, subjects to have difficulties in using electronic healthcare services, which potentially broadens digital divide/exclusion [11, 12].

Alternatively, one-way text messaging from a physician to a patient might be easier to use for the patient since it does not usually require advanced technological skills. This would be an important topic to be investigated further in future studies. In our study, we could not extract the use of text messaging and the use of web-based messaging in the statistical data. In addition, reliable statistical data about electronic communication was available only from 2019 onwards, which is a relatively short follow-up period. In further studies it might be interesting to analyse data from a longer timeperiod to investigate the usage of electronic services and BP levels of patients in primary healthcare more thoroughly.

In the primary healthcare centres of the city of Turku, the proportion of hypertensive subjects with diabetes, coronary artery disease or atrial fibrillation decreased slightly during the follow-up period of our study. In addition, office visits to GPs by hypertensive subjects with coronary artery disease or atrial fibrillation decreased by 15% between 2019 and 2020 (during the COVID-19 pandemic).

The underlying reason might be that the COVID-19 pandemic created disparity in the healthcare services in the most vulnerable hypertensive subgroups.

### Limitations of the study

The cut-off dates defining a year were from 1 January to 31 December. We had access to data only at annual intervals: 2019, 2020, 2021 and 2022. In this current study it was not possible to extract data at shorter intervals, e.g. monthly or weekly, which would have given more detailed information about utilisation of primary healthcare services. We could only assess changes in the number of subjects who contacted primary healthcare units, but we did not have any robust evidence of whether the prevalence of hypertensive patients in the population decreased between 2019 and 2020 or not. Our results are derived only from primary care units of the city of Turku and we did not have access to other healthcare data (secondary healthcare, occupational and private health services) of the inhabitants. This may cause selection bias in our results.

Due to the rapidly aging population of Finland, the aged are the most common users of primary healthcare services. Thus, the average age of the study subjects is higher than in other healthcare facilities. This limits generalisation of our results to other health services and to many other countries. We used only ICD-10 code I10 for defining hypertension. Thus, it is possible that we missed some cases of secondary hypertension but these are a small minority in a primary care population. Unfortunately, it is was not possible to extract this information from the database.

Our primary measures, i.e. GP visits, telephone contacts and electronic communication, are only surrogate endpoints, while the potential underlying main outcome could be extracted from morbidity and mortality data. Unfortunately, in this study we did not have access to BP levels, morbidity or mortality data, nor did we have data on contact patterns among people without hypertension.

In addition, a risk stratification on hypertension severity would have brought more insight to understanding the healthcare service use, but unfortunately, we did not have the possibility to extract, for example, BP measurement data or contact patterns of subjects with hypertension from the database. In addition, we did not have any data on possible hospitalization on hypertensive patients.

In Table III we examined the factors associated with healthcare service use. The models explained between 11% and 14% of office visits, which is, at best, modest.

## Conclusions

In conclusion, the overall utilisation of electronic healthcare services increased during and after the COVID-19 pandemic in primary healthcare in the city of Turku, Finland. This trend was associated with increasing number of office visits to GPs. However, multimorbid (diabetes, coronary artery disease or atrial fibrillation) patients with hypertension attended a GP’s office less frequently than before the pandemic. Even though the present study lacks a cause-and-effect design, it suggests that digital technologies may create structural inequality in access to and availability of primary healthcare services.
